# An assessment of transmission dynamics via time-varying reproduction number of the second wave of the COVID-19 epidemic in Fiji

**DOI:** 10.1098/rsos.220004

**Published:** 2022-08-31

**Authors:** Rajnesh Lal, Weidong Huang, Zhenquan Li, Swastika Prasad

**Affiliations:** ^1^ School of Mathematical and Computing Sciences, Fiji National University, Lautoka, Fiji; ^2^ TD School, University of Technology Sydney, Ultimo, New South Wales 2007, Australia; ^3^ School of Computing and Mathematics, Charles Sturt University, Thurgoona, New South Wales 2640, Australia; ^4^ 9 Satya Place, Lautoka, Fiji

**Keywords:** COVID-19, time-varying reproduction number, Fiji, EpiEstim

## Abstract

This study involves the estimation of a key epidemiological parameter for evaluating and monitoring the transmissibility of a disease. The time-varying reproduction number is the index for quantifying the transmissibility of infectious diseases. Accurate and timely estimation of the time-varying reproduction number is essential for optimizing non-pharmacological interventions and movement control orders during epidemics. The time-varying reproduction number for the second wave of the pandemic in Fiji is estimated using the popular EpiEstim R package and the publicly available COVID-19 data from 19 April 2021 to 1 December 2021. Our findings show that the non-pharmacological interventions and movement control orders introduced and enforced by the Fijian Government had a significant impact in preventing the spread of COVID-19. Moreover, the results show that many restrictions were either relaxed or eased when the time-varying reproduction number was below the threshold value of 1. The results have provided some information on the second wave of the COVID-19 pandemic that could be used in the future as a guide for public health policymakers in Fiji. Estimation of time-varying reproduction numbers would be helpful for continuous monitoring of the effectiveness of the current public health policies that are being implemented in Fiji.

## 1. Introduction

Every country around the globe has been affected by the pandemic caused by coronavirus disease. The coronavirus disease 2019, also known as COVID-19, is a pandemic caused by a novel pathogen called SARS-CoV-2 that started in Wuhan, China, in December 2019 [1]. Several nations are still dealing with the COVID-19 outbreak. As of 30 November 2021, 261 435 768 confirmed COVID-19 cases were reported worldwide, with 5 207 634 COVID-19 related deaths [2].

On 19 March 2020, Fiji reported its first COVID-19 case [3]. Subsequent local transmission cases were confirmed on the 21st, 23rd, 24th and 25th of the same month. The first death resulting from COVID-19 in Fiji was recorded on 31 July 2020 [4]. Approximately after 12 months, Fiji faced the second pandemic wave and recorded its first public transmission of the deadly virus in Nadi town on 19 April 2021. A day later, on 20 April 2021, there was a mass community transmission of COVID-19 cases in Lautoka city [3]. The Delta variant of SARS-CoV-2 was identified as the major reason for the breakthrough infections during the second wave of COVID-19 in Fiji [5]. As of 30 November 2021, Fiji reported 52 506 confirmed COVID-19 cases with 696 deaths [3]. The cumulative confirmed 52 606 cases constitute approximately 99.9% of the confirmed cases after 20 April 2021, i.e. from the second wave of the outbreak in Fiji. Similarly, more than 97% of the deaths occurred after 20 April 2021. Fiji’s population is approximately 900 000. Viti Levu and Vanua Levu, the two main islands, are home to about 87% of the entire population [6]. The more populous island, Viti Levu, has had a much greater reported number of COVID-19 infected cases than Vanua Levu.

Monitoring and assessing the progress of an epidemic outbreak becomes a vital exercise that guides the public health policymakers and government of a given country to make timely decisions. Progress of an epidemiology, including its dynamics, can be obtained from the reported number of confirmed infected cases, recovered cases and death cases [7]. However, in some cases, delays from infection to symptom onset, delays in diagnostic testing including results, delays in symptom onset and admission to clinics and hospitals, and delays in recording and reporting can all obscure the dynamics of epidemiology.

The reproduction number (*R*_*t*_) represents an estimated average number of secondary infected cases that an infected person directly produces over the course of his/her infectious period [8]. The dynamics and progress of a pandemic such as COVID-19 can be monitored by estimating changes in the time-varying (effective) reproduction number. This approach possesses some advantages compared to monitoring using the reported numbers of new or symptomatic cases since estimates of *R*_*t*_ reflect changes in the intensity of the virus transmission [7]. The delays in disease progression will result in a decrease or an increase in the reported number of new cases for a certain period after the virus’s transmissibility has increased or reduced, respectively. The delays mentioned above can be accounted for by monitoring the reproduction number, which can reveal variations in transmissibility that are undetected from the reported number of new cases [7]. The estimates of *R*_*t*_ can also be employed to evaluate and quantify a government’s control interventions and public health regulations in reducing virus transmission [9–12].

The effectiveness of control interventions cannot be evaluated alone from the reported number of new cases since the infected number of cases may still be escalating while there is a decline in transmission [7]. Hence, it follows that tracking of the reproduction number is an important task even after when the control interventions are relaxed.

The reproduction numbers may be estimated in real-time using two different approaches [8,13]. First, epidemiological models, such as ‘Susceptible-Infectious-Removed’ (SIR) or its extensions, e.g. Susceptible-Exposed-Infectious-Recovered, can be estimated following a model-implied construction of time-series reproduction number [14–16]. Second, reproduction numbers can be estimated using an approach that exploits information about a disease’s serial interval (i.e. the difference between the time of symptom onset of a primary case and the onset of the symptoms of the corresponding secondary cases) [17–19].

Recently, the SIR model has been applied to study and assess the spread of the COVID-19 disease and predict the number of infected, removed and recovered populations and deaths in the communities [15,20,21]. Arroyo-Marioli *et al.* [15] used the estimated time-varying growth rate of the COVID-19 cases to estimate the time-varying effective reproduction number of the coronavirus disease. The time-varying reproduction number and disease transmission rate were then employed by the SIR model in tracking the dynamics of COVID-19 around the world. The estimated time-varying reproduction number was then used to assess the effectiveness of non-pharmaceutical interventions in a sample of 14 European countries. Libotte *et al.* [20] employed the SIR model to simulate the dynamic behaviour of COVID-19 considering real data from China. Cooper *et al.* [21] used the SIR model to investigate the spread of COVID-19 disease and estimated the disease trend in various communities, including China, South Korea, India, Australia, the USA and Italy.

Constructing reproduction numbers using epidemiological models demands inverse modelling where model parameters are estimated using optimization algorithms, including data assimilation techniques [15,22–24]. Recently, the popular open source R software package EpiEstim has extensively been used to estimate and evaluate the time-variant (or instantaneous) reproduction numbers of the ongoing COVID-19 epidemic for different countries, for example, [7,10–12,19,25–28].

In this study, we used the open source software package (EpiEstim) and the publicly accessible COVID-19 data to estimate the time-varying reproduction number corresponding to the second wave of the pandemic in Fiji. Our research objective is to determine the effect of the Fijian Government’s response on the COVID-19 epidemic. As a post hoc exercise, we estimated time-varying reproduction numbers to quantify and evaluate the effect of non-pharmacological interventions (policies) implemented by the Fijian Government. Moreover, we discuss the observed associations between the Fijian Government’s control interventions and the estimated time-varying reproduction numbers during the second wave in Fiji. While this study aims to provide some knowledge about the epidemiology of the virus in Fiji, the study’s findings can be used as a reference in future studies pertaining to COVID-19 in Fiji.

The rest of the section of the paper is as follows: we first describe the model EpiEstim used to obtain estimates of the time-varying reproduction number of the COVID-19 pandemic in Fiji. Next, the result section presents the estimated time-varying reproduction number for the second wave of the COVID-19 pandemic. Finally, we discuss the results, examine the estimated time-varying reproduction number with non-pharmacological interventions and movement control orders in Fiji, and end with the conclusion.

## 2. Material and method

This section describes the data sources and processing, R software package EpiEstim and model parameters for estimating time-varying reproduction numbers (*R*_*t*_). A schematic diagram is also provided to illustrate an overview of the EpiEstim model inputs that are combined to generate the estimates of *R*_*t*_.

### 2.1. Data

The publicly available data for the daily new confirmed COVID-19 cases was obtained from the official website of Fiji’s Ministry of Health and Medical Services (MOHFiji) (https://www.health.gov.fj/covid-19-updates/) [3]. Our analysis used the daily time-series data reported from 19 April 2021 (*t* = 1) to 1 December 2021 (*t* = 227). The estimation of time-varying reproduction number using EpiEstim requires the incidence data consisting of daily counts of onset of symptoms. The reporting delay for a case is defined as the time lag in days between the date of onset and the date of reporting. Reporting delays arise from several factors that include delays in case detection, delays in seeking medical care, delays in diagnostics and testing, and delays in processing and recording data in official statistics. A previous study found that learning about reporting parameters, including delay patterns and structure, improves the estimation of reproduction numbers [29]. However, for this study, the only information available was the dates of the report. Hence, the estimated reporting delay was assumed to remain constant over time. The time delay from symptom onset to reporting, which was used for back calculating symptom onset date, was assumed to be 5 days [30,31]. We adjusted the COVID-19 epidemic curve of cases by an assumed fixed reporting delay of 5 days. This implies that the reported dates are shifted backwards by 5 days.

### 2.2. Estimating the time-varying reproduction number, *R*_*t*_

To fit a model for estimating the *R*_*t*_ from the daily number of new confirmed cases (incident cases), *i*_*t*_, the software EpiEstim R package v. 2.2.4 was used [9,17]. EpiEstim, which is optimized for estimating time-varying reproduction numbers, is publicly available at https://cran.r-project.org/web/packages/EpiEstim/index.html.

The *R*_*t*_ measures the number of secondary cases that arise from a person exhibiting signs and symptoms at a specific time, assuming that the conditions stay the same beyond that time. Therefore, *R*_*t*_ is widely used to assess the instantaneous transmissibility of the virus [7]. Apart from its popularity, EpiEstim was preferred for this study because it requires lesser computational time than other similar packages/software [17].

The computation of time-varying reproduction number requires knowing the probability distribution of the *generation time*, i.e. the time interval between a primary infector’s infected date and its consecutive infected date of a secondary case resulting from the primary case [17,32]. In practice, the exact generation time is usually difficult to determine as the infected are seldom identified before the appearance of symptoms. As such, generation time is approximated using the difference between the time of symptom onset of a primary case and the onset of the symptoms of the corresponding secondary cases, which is called a *serial interval* [17,33,34].

Cori *et al.* [17] proposed a Bayesian estimator that considers the unpredictability in the onset of infections, including variations in the serial interval. The authors noted that the expectation of the daily incidence cases (*i*_*t*_) at time *t* is $E[i_{t}]=R_{t}\sum_{s=1}^{t}i_{t-s}\;\omega_{s}$, where *R*_*t*_ is the time-varying reproduction number and *ω*_*s*_ is the probability distribution function of the infectivity profile at time *s*. For an ideal situation in which times of infection are known, the infectivity profile *ω*_*s*_ may be approximated by the distribution of the generation time. However, as the serial interval distribution often approximates the generation time distribution, the estimator is applied to data consisting of daily counts of onset of symptoms where *ω*_*s*_ is approximated by the serial interval distribution [17]. Assuming a gamma prior distribution Γ(a,b) for *R*_*t*_, it follows that an analytical expression of the posterior distribution of *R*_*t*_ can be obtained [17]. The reproduction number is estimated as

2.1 $$R_{t}=\frac{i_{t}}{\sum_{s=1}^{t}i_{t-s}\;\omega_{s}},\;t=1,2,\ldots ,T,$$

where *T* is the final time point of the series data of the incidence cases. Owing to reporting errors, the resulting reproduction number can be highly variable at smaller time steps. Hence, to obtain smother estimates, *R*_*t*_ is calculated in a time window of size *τ* ending at time *t*, assuming that *R*_*t*_ remains constant within that time window. The above model is implemented using the estimate_R function from the EpiEstim package.

**Figure 1.**
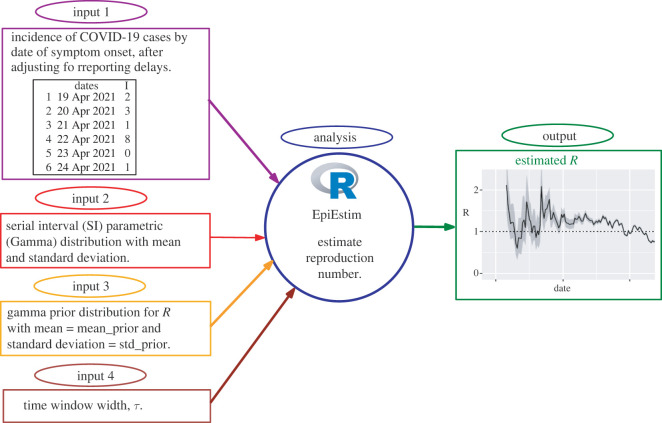
A schematic illustrating how the inputs (data and model parameters) are combined to generate the estimates of the time-varying reproduction number using the EpiEstim R package.

Figure 1 provides an overview of the EpiEstim package inputs (data and model parameters) that are combined to generate the output, i.e. estimates of the time-varying reproduction number.

### 2.3. Model parameters

The goal is to make as few assumptions as possible to make reasonable forecasts without relying on the assumptions being correctly specified. We were unable to explicitly compute the parameters of the serial interval distribution because we did not have access to more specific infector-infected data pairs (e.g. contact tracing patient data). The serial interval distribution is the most crucial assumption to make. Ryu *et al.* [35] investigated the serial interval and transmission dynamics during SARS-CoV-2 Delta variant predominance in South Korea, and reported the estimated mean and standard deviation of the serial interval distribution.

For this study, we assumed a discrete gamma-distributed SI for SARS-CoV-2 with mean of 3.6 days and a standard deviation (s.d.) of 4.9 days following the reported results of [35]. Furthermore, we used the findings of a recent research by Imai *et al.* [36] for the basic reproduction number (*R*_0_), which was calculated using data from the early stages of the COVID-19 epidemic in Wuhan. Following [36], we set the gamma prior for *R*_*t*_ with a mean (mean_prior) of 2.6 and standard deviation (std_prior) 2. Using the reporting delay adjusted incidence curve, the *R*_*t*_ was estimated with EpiEstim on sliding weekly windows (*τ* = 7).

Three sensitivity analyses were conducted to quantify the effect of model parameter changes on estimated *R*_*t*_ values. Zhang *et al.* [37] studied the transmission dynamics of an outbreak of the COVID-19 Delta variant in Guangdong Province, China and reported the estimated mean and s.d. of serial interval distribution as 2.3 days and 3.4 days, respectively. Similarly, a study by Li *et al.* [38] on the transmission and containment of the SARS CoV-2 Delta variant in Guangzhou, China, reported the estimated mean and s.d. of serial interval distribution as 4.24 days and 3.95 days, respectively. We used the mean and SD values from [37,38] for the first two sensitivity analyses. All other settings were kept the same. EpiEstim assumes a default gamma prior for *R*_*t*_ with a mean_prior and std_prior of 5, ensuring that when there are few infections, the prior becomes disproportionately weighted relative to the data [39]. The third sensitivity analysis considered using the default gamma prior for *R*_*t*_.

All data analysis and simulations were performed using the software R v. 4.1.2 (https://cloud.r-project.org/).

### 2.4. Model validation

To assess the accuracy of the estimated *R*_*t*_ and to numerically quantify the goodness of fit between the observed and modelled number of cases, the coefficient of determination *r*^2^ was computed using

2.2 $$r^{2}=1-\frac{\sum_{t=1}^{T}{\left(i_{t}-\hat{i}{\;}_{t}\right)}^{2}}{\sum_{t=1}^{T}{\left(i_{t}-\bar{i}{\;}_{t}\right)}^{2}},$$

where *i*_*t*_ is daily incidence cases (observed) at time *t*, ${\hat{i}}_{t}$ is the modelled number of cases at time *t* and ${\bar{i}}_{t}$ is the average daily incidence cases. To compute the modelled number of cases ${\hat{i}}_{t}$, we first determined the total infection potential across all infected individuals at time *t*, $\Lambda_{t}$ computed as [9]

2.3 $$\Lambda_{t}=\sum_{s=1}^{t}i_{t-s}w_{s},\;t=1,2\ldots ,T.$$

We employed the overall_infectivity function from the EpiEstim package to compute $\Lambda_{t}$. The modelled number of cases was then computed as ${\hat{i}}_{t}=R_{t}\Lambda_{t}$.

## 3. Results

**Figure 2.**
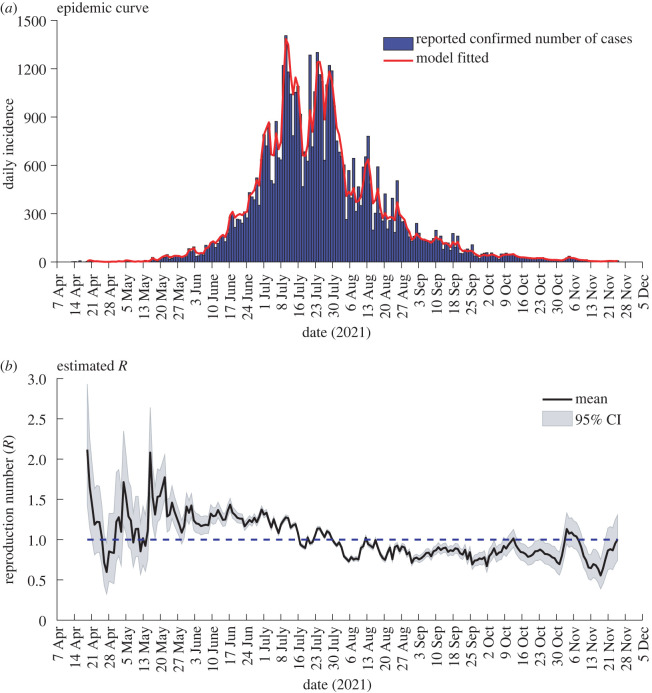
Outputs from *EpiEstim*. (*a*) The epidemic curve shows the observed daily number of new COVID-19 cases (in blue) and the model fitted number of cases (in red) by the assumed date of symptom onset. (*b*) Estimates of the time-varying reproduction number *R*_*t*_ over sliding weekly windows. The solid line is the mean value and the shaded areas around the mean represent 95% CI. *R*_*t*_ is less than 1 below the horizontal dotted line, indicating a slowing epidemic in which new infections are not increasing.

The output (plots) from EpiEstim is shown in figure 2. Figure 2*a* gives the observed (in blue) and model fitted (in red) daily number of COVID-19 cases by the assumed date of symptom onset. The fitted daily numbers of cases agree well with the observed ones. The coefficient of determination value of *r*^2^ = 0.9674 further confirms a good quality fit of the modelled number of cases. It is noted that the daily incidence was highest on 11 July 2021, with 1405 new cases. The number of new daily incidence gradually decreased from 1 August 2021. Figure 2*b* shows the transmissibility of COVID-19 in Fiji estimated based on *R*_*t*_, where the solid line is the mean *R*_*t*_ and the shaded area around the mean represents a 95% credible interval (CI).

An increase in *R*_*t*_ suggests increasing transmissibility of the virus. The epidemic will gradually disappear if the *R*_*t*_ values remain consistently below the threshold value of 1. In figure 2, a high *R*_*t*_ = 2.11 (95% CI: 1.44–2.93) at the beginning of the second wave is due to a mass community transmission of the virus in Lautoka city [3]. The two peaks *R*_*t*_ = 1.71 (95% CI: 1.17–2.35) on 5 May 2021 and *R*_*t*_ = 2.08 (95% CI: 1.59–2.64) on 16 May 2021, are due to the SARS-CoV-2 virus spreading to other regions of the nation, including Lami, Suva and Nausori in Viti Levu’s central division [3,40].

The *R*_*t*_ values were greater than 1 from 17 May 2021 to 17 July 2021, with *R*_*t*_ fluctuating between 1.08 (95% CI: 1.06–1.11) and 2.08 (95% CI: 1.59–2.64). This reflected a high transmission of the disease, and as a result, the number of new confirmed cases continued to rise in the same time span, as seen in figure 2*a*.

With strict measures, including non-pharmacological interventions (NPIs) and movement control (MCOs) orders, Fiji managed to curb the exponential increase in the COVID-19 cases. This caused the *R*_*t*_ values to decrease slowly, and eventually, between 1 August 2021 and 4 November 2021, *R*_*t*_ values were either less than 1 or very close to 1. As depicted in figure 2*a*, the decrease in the number of new cases between 1 August 2021 and 4 November 2021 can be attributed to the *R*_*t*_ values being less than 1 in the same time interval. Moreover, the results show that the virus’s transmission was under control between 1 August 2021 and mid-October.

Some relaxations of restrictions implemented since mid-October [40,41] caused a spike on 5 November 2021. Nevertheless, the transmission of the virus was well under control since *R*_*t*_ values had already decreased significantly and remained around 1 or less.

**Figure 3.**
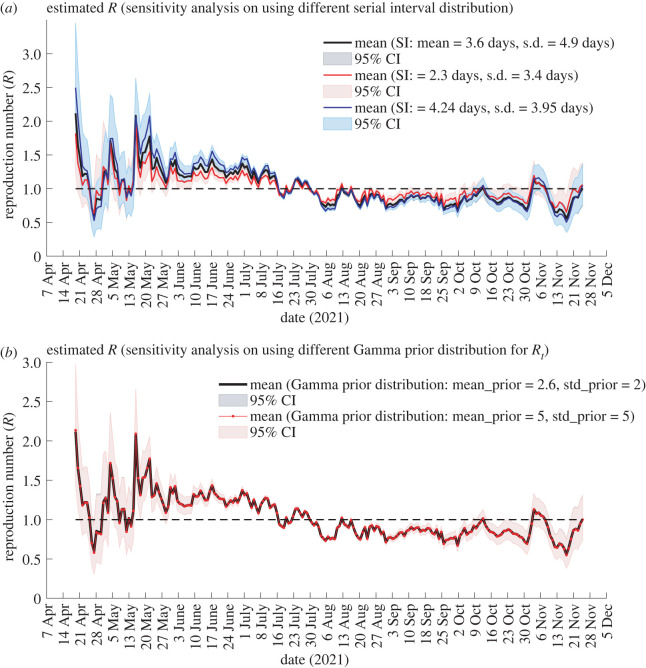
Sensitivity analysis. Estimates of time-varying reproduction number considering three different serial interval distributions (*a*) and considering two different gamma prior distributions for *R*_*t*_ (*b*). The solid line is the mean value and the shaded areas around the mean represent 95% CI. For all the cases, *R*_*t*_ was estimated on sliding windows of width *τ* = 7 days.

Figure 3 depicts sensitivity analysis results using different serial interval distributions (*a*) and using different gamma prior distributions for *R*_*t*_ (*b*). In figure 3*a*, we note that the plots share similar characteristics. The sensitivity analyses showed that the estimate of *R*_*t*_ is influenced by the mean and standard deviation of serial interval. The longer SI resulted in higher *R*_*t*_ estimates. However, the impact of different serial interval distributions was small, and the difference in profiles is supported by the 95% CI. In figure 3*b*, we notice that a default gamma prior for *R*_*t*_ yielded similar result. The estimates of *R*_*t*_ were not sensitive to the choice of gamma prior for *R*_*t*_.

## 4. Discussion

In Fiji, like most countries, various control measures including NPIs and MCOs were implemented to contain and prevent the spread of the COVID-19 virus. The five broad categories of measures included (i) *lockdown*: either partial or full lockdowns of cities, including the containment areas/zones, (ii) *movement restrictions*: such as border checks, checkpoints within the country, imposing curfews, and suspension of domestic and international flights, (iii) *social distancing*: closure of businesses and limiting public services, banning or limiting public gatherings, and closure of schools, (iv) *public health measures*: awareness campaigns, health screenings in airports, isolation and quarantine policies, mass population testing, mandatory face mask wearing, use of careFIJI mobile app for contact tracing, compulsory vaccination requirement of employees, and (v) *governance and socioeconomic measures*. Table 1 summarizes the timeline of NPIs and MCOs in Fiji from 19 April 2021 to 1 December 2021 [3,40,41].

**Table 1.** Timeline of the non-pharmacological interventions and movement control orders in Fiji. Source [3,40,41].

date	interventions and movement control in Fiji from 19 Apr 2021 to 1 Dec 2021.
19 Apr	Lockdown of Nadi and Lautoka cities. Residents can leave home for critical reasons only. Closure of schools and non-essential business. Restaurants to operate for delivery and takeaway services. Most international commercial flights remain suspended. The night curfew hours from 23.00 to 4.00. Mandatory use of the government’s careFIJI mobile application contact tracing. Prohibition of public meetings and gatherings for religious, social, sports and cultural activities. Funerals to take place with up to 20 persons.
26 Apr	Movements are restricted in Viti Levu’s Central Division, including Suva, Lami and Nausori.
3 May	Public transportation within containment zones runs at half capacity. Suspension of all international commercial and repatriation flights. Foreign nationals are not permitted to enter Fiji unless they have received formal permission from immigration officials. All entrants into Fiji are subject to a mandatory health inspection and a 14-day quarantine.
21 May	Mandatory facemasks wearing, temperature checks, 2-m distancing and use of government’s careFiji mobile application continues. An 18.00 to 4.00 curfew island wide on Viti Levu.
26 May	14-day lockdown of the capital city, Suva.
3 July	Restrictions on movement in the Central Division of Viti Levu (a night curfew from 18.00 to 4.00). The remaining of Viti Levu has a night curfew from 20.00 to 4.00.
8 July	Announcement of ‘no jab, no job’ policy.
26 July	Freedom of movement severely restricted. A nighttime curfew from 18.00 to 4.00 in the Western Division and the city of Suva.
27 Aug	Lockdown of a village in Labasa, Vanua Levu.
17 Sep	Eased business and travel restrictions across Fiji. Viti Levu-night curfew hours reduced to 21.00 to 4.00. Public transportation can run at 70% capacity. Ease of restriction on public gathering with a maximum of 30 people and mandatory social distancing in public places.
20 Sep	COVID-19 vaccine rolled out to children between 15 and 17 years.
4 Oct	Houses of worship, tertiary institutions and workplaces opens up to 70% capacity to fully vaccinated adults.
7 Oct	New night curfew hours from 22.00 to 4.00.
13 Oct	Shortened night curfew hours from 23.00 to 4.00 daily. Indoor venues can hold gatherings of vaccinated people up to 80% of their capacity. Mandatory social distancing in public places.
1 Nov	Opening of schools for Years 12 and 13 students.
11 Nov	Fiji lifted an entry ban for fully vaccinated returning residents, permit holders, diplomats and other approved travellers from 27 travel partner locations with mandatory 3-day quarantine and COVID-19 test on day two of quarantine. Officials permit fully vaccinated people to attend gatherings of any size outdoors or up to 80% capacity indoors.
17 Nov	All quarantine measures were lifted for domestic travel.
18 Nov	90% of target individuals were fully vaccinated. Curfew hours moved from midnight to 4.00.
28 Nov	Strengthening of strict border control measures in response to newly detected coronavirus variant (Omicron).
1 Dec	Fiji permits tourist travel for fully vaccinated people from Travel Partner locations.

**Figure 4.**
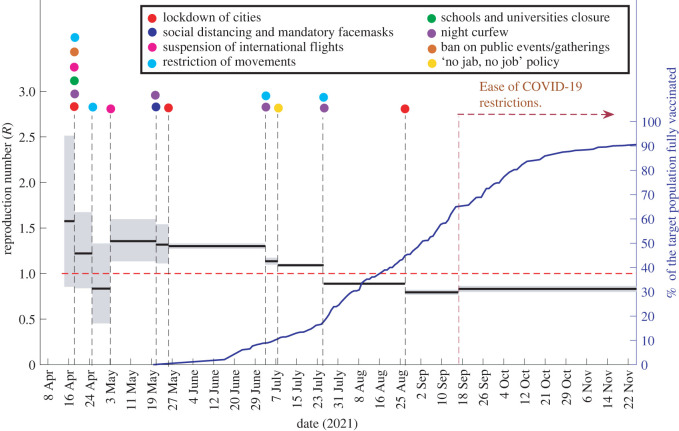
The estimated stepwise *R*_*t*_ between dates (table 1) when NPIs and MCOs were implemented. The coloured dots indicate the implemented NPIs and MCOs. The blue line represents the cumulative percentage of the eligible population in Fiji who received two doses of COVID-19 vaccines [3].

The stepwise *R*_*t*_ between each date when an intervention was implemented is computed using the estimate_R function by specifying the vectors t_start and t_end. t_start and t_end are the vectors of positive integers giving each window’s starting and ending times, respectively, over which the reproduction number will be estimated. Figure 4 exhibits the estimated stepwise *R*_*t*_ between dates (table 1) when NPIs and MCOs were implemented. The coloured dots in figure 4 indicate the implemented NPIs and MCOs. The blue line in figure 4 represents the cumulative percentage of the eligible population in Fiji who were completely vaccinated, i.e. got two doses of COVID-19 vaccinations [3].

The results shown in figure 4 suggest that the NPIs and MCOs implemented at the beginning (on 19 April 2021) were effective in mitigating the transmissibility of the disease. The interventions and movement control policies included lockdown of cities, night curfews, closure of schools and universities, suspension of international flights, ban on public gatherings and restriction of movements. During the pandemic’s initial phase, the mean *R*_*t*_ estimates demonstrated a high value of 1.58 (95% CI: 0.86–2.51), which was reduced to 1.22 (95% CI: 0.84–1.67) after the implementation of the NPIs and MCOs. A further restriction of movements imposed in Viti Levu’s Central Division on 26 April 2021 resulted in a significantly lower mean *R*_*t*_ estimate below 1, i.e. 0.83 (95% CI: 0.45–1.33). However, the mean *R*_*t*_ estimate from 3 May 2021 to 21 May 2021 increased to 1.36 (95% CI: 1.13–1.60). This increase can be attributed to the allowance of public transport services inside the containment zones.

Our results further support the protective benefits of public health measures such as 2-m physical distancing, mandatory facemasks wearing and implementation of a night curfew. This is evident from the decrease of the mean *R*_*t*_ estimates from 21 May 2021 to 8 July 2021. On 8 July 2021, the Fijian Government initiated the ‘no jabs, no job’ policy [42]. In effect of this policy, there was an increase in the vaccination rate, as seen by the percentage values in figure 4. On 26 July 2021, freedom of movement was severely restricted, and a nighttime curfew was implemented from 18.00 to 4.00 in the western division and the city of Suva. Consequently, from 26 July 2021 to 27 August 2021, the mean *R*_*t*_ estimates were lowered to 0.89 (95% CI: 0.88–0.90). The mean *R*_*t*_ estimates remained below the threshold 1 after 27 August 2021. Moreover, we notice a significant increase in the vaccination rate after 27 August 2021. Many restrictions were either relaxed or eased after 17 September 2021.

We note that some of the changes in control policies were implemented due to an increase in vaccination coverage. For example, on 13 October 2021, 80% of the target population were fully vaccinated, and as a result, the night curfew hours were shortened, and gatherings were permitted indoors in people’s homes. Later, on 18 November 2021, the night curfew hours were shortened because 90% of the target populations were fully vaccinated [40].

Our results show that timely implementation of different NPIs and MCOs by the Fijian Government resulted in significant decelerations of COVID-19 progress. The enforcement of lockdown of cities, social distancing and mandatory facemasks, restriction of movements (including implementation of curfew), the ban on public gatherings, and closure of schools and universities were associated with the decrease in *R*_*t*_ and the number of daily incidence cases. Our findings are consistent with previous studies that have reported that policies, such as the closure of schools, bans on large public gatherings, lockdowns and MCOs, were associated with a significant reduction in *R*_*t*_ and the incidence of COVID-19 cases [43,44].

We note that the estimates of *R*_*t*_ in this study are based on the number of the reported cases and the choice of model parameters such as the distribution for the SI. While the public health decision-making process can be aided by reviewing the association between measures (NPIs and MCOs) and the estimated *R*_*t*_, the insights and findings from our analysis should be regarded with caution. As such, we present two apparent limitations of this study. First, we ignored the delays and errors in the reported number of new cases. The model is data-driven, and estimated *R*_*t*_ are prone to errors and reporting rates. As the number of new cases is affected by testing capacity, the estimated initial *R*_*t*_ is influenced by testing frequency, contact tracing time and the rate of reporting of the new cases [45]. The second limitation concerns the choice of parameter values. We fully agree that generation time and SI may differ from other countries due to different NPIs and MCOs, and testing capabilities. Unfortunately, we did not have access to infector-infected data pairs to estimate SI.

Future work includes the estimation of *R*_*t*_ of COVID-19 in Fiji using the SIR model to assess the effectiveness of public policy interventions. Comparisons of results obtained with EpiEstim and the SIR model could be made. Other models, such as the modified SIR model that accounts for vaccination [46–48] will be considered in future studies.

## 5. Conclusion

This study estimated the *R*_*t*_ of the second wave of the COVID-19 pandemic using the data on confirmed cases in Fiji. Given that the estimated *R*_*t*_ may be biased, it is necessary to regard these results with caution. However, the model shows that the NPIs and MCOs introduced and enforced from 19 April 2021 to 1 December 2021 by the Fijian Government had a significant impact in preventing the spread of COVID-19. We found that the COVID-19 transmission in Fiji had the strongest association with the lockdown of cities, social distancing and mandatory facemasks, restriction of movements (including implementation of curfew), closure of schools and universities and the ban on public gatherings. Moreover, we showed that timely implementation of the control measures resulted in the reduction of *R*_*t*_ and the incidence of COVID-19 cases. The results have provided some information on the second wave of the COVID-19 pandemic that could be used in the future as a guide for public health policymakers in Fiji. As the *R*_*t*_ values were strongly influenced by the NPIs and MCOs measures, the results can be used to analyse and evaluate the effectiveness of NPIs and MCOs interventions. Moreover, with COVID-19 cases still present in Fiji, an estimation of *R*_*t*_ would be helpful for continuous monitoring of the effectiveness of the current public health policies implemented in Fiji.

## Data Availability

Data and relevant code for this research work are stored in GitHub: https://github.com/drrajneshlal/Fiji_COVID and have been archived within the Zenodo repository: https://doi.org/10.5281/zenodo.6596312 [49].
